# Automated sparse feature selection in high-dimensional proteomics data via 1-bit compressed sensing and K-Medoids clustering

**DOI:** 10.1186/s12859-025-06193-2

**Published:** 2025-07-01

**Authors:** FuDong Wen, Yue Su, Dan Liu, YuPeng Wang, MeiNa Liu

**Affiliations:** Department of Biostatistics, Public Health College, Harbin Medical University, Harbin City, 150081 Heilongjiang Province China

**Keywords:** Feature selection, Classification, Compressed sensing, K-Medoids clustering, Proteomics

## Abstract

**Background:**

High-dimensional proteomics data present significant challenges in biomarker discovery due to technical noise, feature redundancy, and multicollinearity. Current feature selection methods, including filter, wrapper, and embedded approaches, struggle with stability, sparsity, and computational efficiency. To address these limitations, we propose Soft-Thresholded Compressed Sensing (ST-CS), a hybrid framework integrating 1-bit compressed sensing with K-Medoids clustering. Unlike conventional methods relying on manual thresholds, ST-CS automates feature selection by dynamically partitioning coefficient magnitudes into discriminative biomarkers and noise.

**Results:**

Evaluations on simulated and real-world proteomic datasets demonstrated ST-CS’s superiority in feature selection capability and classification performance. In simulations, ST-CS achieved feature selection robustness with balanced sensitivity (> 80%) and specificity (> 99.8%), reducing false discovery rates (FDR) by 20–50% compared to Hard-Thresholded Compressed Sensing (HT-CS). Additionally, it attained superior F1 scores and Matthews Correlation Coefficients (MCC), outperforming HT-CS, LASSO, and SPLSDA in identifying true biomarkers while suppressing noise. For classification performance, ST-CS surpassed all methods in the area under the receiver operating characteristic curve (AUC) across varying noise levels while maintaining sparsity. Applied to Clinical Proteomic Tumor Analysis Consortium (CPTAC) datasets, ST-CS matched HT-CS’s classification accuracy (AUC = 97.47% for intrahepatic cholangiocarcinoma) but with 57% fewer selected features (37 vs. 86), demonstrating its dual strength in precision biomarker discovery and predictive accuracy. For glioblastoma data, ST-CS achieved higher AUC (72.71%) than HT-CS (72.15%), LASSO (67.80%), and SPLSDA (71.38%) while retaining a parsimonious feature set (30 vs. 58 features for HT-CS). In ovarian serous cystadenocarcinoma, ST-CS further demonstrated its adaptability, attaining superior AUC (75.86%) over HT-CS (75.61%), LASSO (61.00%), and SPLSDA (70.75%) with only 24 ± 5 selected biomarkers. These results highlight ST-CS’s ability to rigorously automate feature selection while balancing classification efficacy, interpretability, and scalability for translational proteomics.

**Supplementary Information:**

The online version contains supplementary material available at 10.1186/s12859-025-06193-2.

## Background

Proteins, as the primary executors of biological functions, play a pivotal role in elucidating molecular mechanisms underlying diseases such as cancer and neurodegenerative disorders. Proteomics, powered by advancements in mass spectrometry, enables systematic profiling of protein expression in human blood, offering unprecedented opportunities for biomarker discovery and clinical diagnostics [1]. Despite these advances, modern medical studies reveal that only a small fraction of detected proteins are biologically relevant to specific pathologies, while the majority represent technical noise or non-causal correlations [2]. This discrepancy underscores the urgent need for robust dimensionality reduction and precise feature selection in high-dimensional proteomic datasets, which are characterized by three key challenges: (1) noise stemming from technical variability in mass spectrometry, (2) high dimensionality where the number of measured protein features vastly exceeds the sample size, and (3) strong inter-protein correlations arising from functional biological networks [3].

Current feature selection methodologies face critical limitations in addressing these challenges. Filter methods (e.g., ANOVA, Pearson’s correlation) rank features via univariate metrics but neglect multivariate interactions [4]. Wrapper approaches, such as genetic algorithms, iteratively optimize feature subsets using predictive models but suffer from prohibitive computational costs in high-dimensional settings [5]. Embedded methods, including the Least Absolute Shrinkage and Selection Operator (LASSO) [6] and elastic net [7], integrate feature selection with model training through regularization. However, LASSO’s aggressive ${\ell}_{1}$-norm shrinkage often discards weakly correlated biomarkers in highly collinear proteomic data, while elastic net’s hybrid ${\ell}_{1}$ and ${\ell}_{2}$ penalties improve stability at the expense of reduced sparsity. Sparse partial least squares discriminant analysis (SPLSDA), though effective in handling multicollinearity through latent variable construction, often retains redundant features due to its aggregation of correlated but non-informative signals, thereby diluting biomarker specificity [8]. Furthermore, SPLSDA requires simultaneous tuning of multiple hyperparameters (e.g., sparsity thresholds and component numbers), which complicates optimization and risks overfitting in high-noise regimes. Compressed sensing (CS) is a signal processing technique that recovers sparse signals from under-sampled measurements. The 1-bit variant of CS simplifies this process by quantizing continuous measurements (e.g., protein intensities) into binary values (+ 1 or −1), effectively reducing noise and computational complexity. This binary quantization aligns with classification tasks where outcomes are often categorical (e.g., diseased vs. healthy), enabling robust sparse signal recovery even in high-noise settings [9]. Yet, conventional 1-bit CS relies on manual thresholding to select features—a process prone to subjectivity and suboptimal generalization.

To overcome these limitations, we propose a novel hybrid framework, Soft-Thresholded Compressed Sensing (ST-CS), which synergizes 1-bit CS with K-Medoids clustering [10] to automate feature selection. Unlike fixed thresholding in traditional 1-bit CS, our method dynamically partitions coefficient magnitudes into discriminative biomarkers and noise via data-driven clustering. This innovation uniquely combines the sparse signal recovery capability of CS with the adaptability of unsupervised learning, addressing two critical gaps: (1) the subjectivity of manual thresholds and (2) the instability of sparse models in collinear, high-noise environments. By enforcing dual ${\ell}_{1}$ and ${\ell}_{2}$ regularization, ST-CS balances sparsity and stability, while K-Medoids clustering objectively separates true signals from noise. Our approach not only enhances specificity and precision in biomarker identification but also preserves computational efficiency, making it scalable for large-scale proteomic studies. The primary objectives of this study are to (1) establish a theoretical foundation for integrating compressed sensing with clustering-based thresholding, (2) rigorously evaluate ST-CS against state-of-the-art methods in simulated and real-world proteomic datasets, and (3) provide an open-source implementation to facilitate translational applications in precision medicine.

## Methods

### Linear decision function

In supervised classification frameworks, the primary objective is to derive a decision boundary that optimally separates two classes (e.g., diseased vs. healthy) based on high-dimensional input features. For proteomic data, where each sample ${{\varvec{x}}}_{i}\in {\mathbb{R}}^{d}$ represents a vector of protein intensities (e.g., mass spectrometry measurements), we employ a linear decision function to quantify the discriminative relationship between features and class labels. Unlike traditional CS frameworks that employ a measurement matrix for dimensionality reduction, the 1-bit CS approach adopted here quantizes decision scores to binary labels. This eliminates the need for explicit linear measurements, focusing instead on recovering sparse coefficients that satisfy sign consistency with observed class labels. Formally, the decision score for the $i$-th sample is computed as:

$${f}_{{\varvec{\omega}}}\left({{\varvec{x}}}_{i}\right)=\langle {\varvec{\omega}},{{\varvec{x}}}_{i}\rangle ={\sum }_{k=1}^{d}{\omega }_{k}{x}_{i,k},\quad i=1,\dots ,n,$$

where, $\varvec{\omega } = (\omega _{1} ,\omega _{2} , \ldots ,\omega _{d} )^{\top} \in {\mathbb{R}}^{d}$ denotes the coefficient vector, with each ${\omega }_{k}$ reflecting the relative contribution of the $k$-th protein to class discrimination; ${{\varvec{x}}}_{i}=({x}_{i,1},{x}_{i,2},\dots ,{x}_{i,d}{)}^{\top }$ encodes the proteomic profile of the $i$-th sample; $\langle \cdot ,\cdot \rangle$ represents the Euclidean inner product, linearly combining weighted feature values into a scalar decision score.

The classifier enforces sign consistency between predicted scores and binary labels ${y}_{i}\in \{-1,+1\}$:

$${y}_{i}={sign}\left({f}_{{\varvec{\omega}}}\left({{\varvec{x}}}_{i}\right)\right),\quad \forall i\in \left\{1,\dots ,n\right\}.$$

Geometrically, ${\varvec{\omega}}$ defines a hyperplane $\langle {\varvec{\omega}},{\varvec{x}}\rangle =0$ in the $d$-dimensional feature space, partitioning samples into two classes:

- Samples with ${f}_{{\varvec{\omega}}}({{\varvec{x}}}_{i})>0$ are assigned to the $+1$ class (e.g., diseased), while those with ${f}_{{\varvec{\omega}}}\left({{\varvec{x}}}_{i}\right)<0$ belong to the $-1$ class (e.g., healthy).
- The magnitude $|{\omega }_{k}|$ quantifies the discriminative importance of the $k$-th protein. Nonzero coefficients $({\omega }_{k}\ne 0)$ identify biomarkers, whereas ${\omega }_{k}\approx 0$ implies irrelevance to classification.
- The sign of ${\omega }_{k}$ distinguishes whether the $k$-th protein is upregulated (positive ${\omega }_{k}$) or downregulated (negative ${\omega }_{k}$) in the $+1$ class relative to the $-1$ class.

*Example *: Consider a coefficient vector ${\varvec{\omega}}=(1.5,-\text{0.3,0},\dots ,0{)}^{\top }$*.* Here, the first protein $({\omega }_{1}=1.5)$ strongly associates with the $+1$ class, the second protein $({\omega }_{2}=-0.3)$ weakly correlates with the $-1$ class, and the third feature $({\omega }_{3}=0)$ is non-informative. A sample with high intensity in the first protein and low intensity in the second would yield a large positive score, aligning with the $+1$ label.

While this linear model offers interpretability through direct biomarker identification, unconstrained optimization in high-dimensional settings risks overfitting due to redundant or noisy features, which necessitates regularization techniques (section "Optimization framework") to enforce sparsity and stabilize coefficient estimates.

### Optimization framework

Building upon the linear decision function defined in section "Linear decision function", we formulate a constrained optimization problem to estimate the coefficient vector ${\varvec{\omega}},$ while addressing the inherent challenges of high-dimensional proteomics data. When the feature dimension $d$ far exceeds the sample size $n \left(d\gg n\right)$, unregularized linear models are prone to overfitting due to the "curse of dimensionality" and noise amplification. To stabilize coefficient estimates and enforce sparsity—critical for identifying a small subset of biologically relevant biomarkers—we integrate dual regularization through ${{\ell}}_{1}$-norm (sum of absolute coefficients) and ${{\ell}}_{2}$-norm (sum of squared coefficients) constraints. This optimization corresponds to the reconstruction phase of the 1-bit CS framework, where sparse coefficients ${\varvec{\omega}}$ are recovered under sign consistency constraints.

The optimization objective balances two competing goals:

1. Maximizing class separation: The term $\sum_{i=1}^{n} {y}_{i}\langle {{\varvec{x}}}_{{i}},{\varvec{\omega}}\rangle$ quantifies alignment between predicted scores and true labels. Maximizing this sum ensures that the decision hyperplane $\langle {\varvec{\omega}},{\varvec{x}}\rangle =0$ effectively discriminates between classes.
2. Regularizing model complexity: The ${{\ell}}_{1}$-norm constraint $\|{\varvec{\omega}}{\| }_{1}\le \sqrt{\lambda }$ promotes sparsity by shrinking irrelevant coefficients to zero, mimicking biological parsimony where few proteins drive class differences. The ${{\ell}}_{2}$-norm constraint $\|{\varvec{\omega}}{\| }_{2}\le 1$ controls multicollinearity among correlated features (common in proteomic networks) and prevents arbitrary coefficient inflation.

Formally, the constrained optimization problem is:

$$\begin{aligned} &{\varvec{\omega}}^{*} = \arg \mathop {\max }\limits_{{{\varvec{\omega}} \in {\mathbb{R}}^{d} }} \mathop \sum \limits_{i = 1}^{n} y_{i} \left\langle {{\varvec{x}}_{i} ,{\varvec{\omega}}} \right\rangle \hfill \\ &{\text{subject to}}\left\| \varvec{\omega} \right\|_{1} \le \sqrt \lambda , \hfill \\ & \quad\quad\quad\quad\,\, \left\| \varvec{\omega} \right\|_{2} \le 1. \hfill \\ \end{aligned}$$

where $\lambda > 0$ is a hyperparameter controlling the sparsity-intensity trade-off. Smaller $\lambda$ tightens the ${{\ell}}_{1}$ constraint, yielding sparser solutions, while larger $\lambda$ relaxes sparsity to retain more features.

*The intuition behind the dual constraints***:** The ${{\ell}}_{1}$-norm induces a “sparse signal” prior, aligning with the biological assumption that only a few proteins are true biomarkers. The ${{\ell}}_{2}$-norm ensures stable coefficient estimates despite feature correlations, acting as a ridge-like stabilizer. Together, these constraints mimic the elastic net regularization within a constrained (rather than penalized) optimization framework. This formulation explicitly avoids the overparameterization issues of unregularized models while maintaining computational tractability. The resulting coefficients ${{\varvec{\omega}}}^{*}$ inherently reflect feature importance, with nonzero entries indicating candidate biomarkers.

*Implementation note***:** The problem is convex and solved numerically using the Rdonlp2 package, which employs sequential quadratic programming. The dual constraints are scaled to ensure comparable regularization effects across datasets.

### Sparse coefficient selection via K-Medoids clustering

The integration of 1-bit CS and K-Medoids clustering operates in two sequential phases. First, the constrained optimization (Section "Optimization Framework") recovers a sparse coefficient vector ${{\varvec{\omega}}}^{*}$, which satisfies the sign consistency required for binary classification. Second, the magnitudes $\left|{{\varvec{\omega}}}^{*}\right|$ are partitioned via K-Medoids clustering, automating the distinction between true biomarkers (large coefficients) and noise (near-zero coefficients). This replaces subjective manual thresholds with an adaptive, data-driven process.

**Fig. 1:**
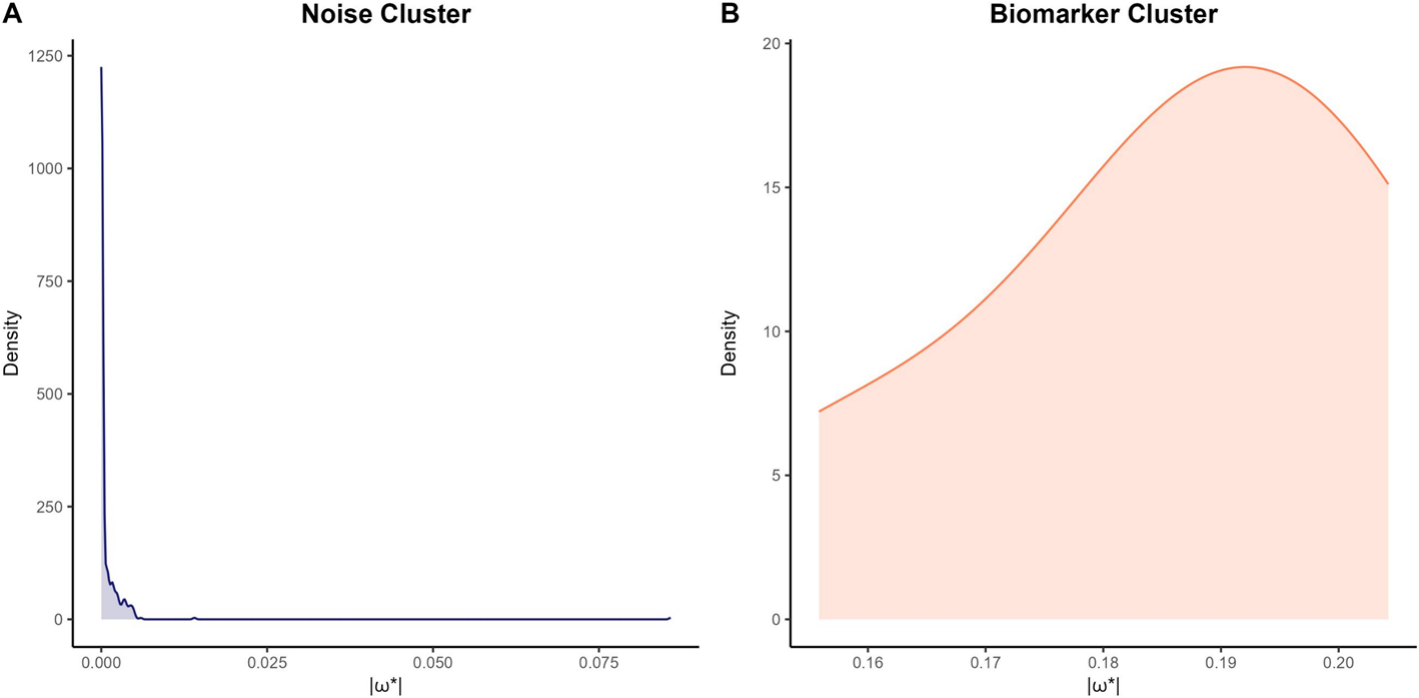
Bimodal distribution of absolute coefficients $\left|{\varvec{\omega}}^{*}\right|$ from Simulation. The kernel density estimate demonstrates a clear bimodal separation: a dense cluster of near-zero coefficients (blue, noise features) and a distinct high-magnitude cluster (red, true biomarkers). This visualization validates our hypothesis that regularization naturally induces a bimodal structure, enabling data-driven thresholding via K-Medoids

The rationale for clustering stems from the bimodal distribution of $\left|{{\varvec{\omega}}}^{*}\right|$ (Fig. 1): coefficients corresponding to true biomarkers tend to exhibit larger magnitudes, while irrelevant features cluster near zero due to regularization. This bimodality arises naturally from the interplay between regularization and sparse signal recovery, enabling automated separation of biomarkers from noise. By partitioning the absolute coefficients into two groups ($K=2$), we automate the identification of a cutoff that optimally discriminates between these two regimes. K-Medoids was selected over K-Means due to its robustness to outliers and flexibility in handling non-spherical data distributions. Unlike K-Means, which uses mean-based centroids sensitive to extreme values, K-Medoids employs actual data points (medoids) as cluster centers. Combined with Manhattan distance, this ensures stable partitioning of coefficient magnitudes, even in high-noise regimes. These properties align with the bimodal structure of $\left|{{\varvec{\omega}}}^{*}\right|$, where true biomarkers and noise exhibit distinct magnitude profiles. The clustering proceeds as follows:

1. *Input Preparation*: Compute the absolute values of the optimized coefficients, ${\varvec{v}}=(|{\omega }_{1}^{*}|,|{\omega }_{2}^{*}|,\dots ,|{\omega }_{d}^{*}|)$.
2. *Cluster Initialization*: The first medoid ${m}_{1}$ is fixed to the maximum absolute coefficient value ($max(\left|{{\varvec{\omega}}}^{*}\right|)$), representing the strongest biomarker signal, while the second medoid ${m}_{2}$ is fixed to the minimum value ($min(\left|{{\varvec{\omega}}}^{*}\right|)$), representing the weakest noise.
3. *Cluster Assignment*: Assign each coefficient ${v}_{j}$ to the nearest medoid using Manhattan distance for outlier resistance:
  $$C_{t} = \left\{ {v_{j} {\text{|arg}}\mathop {min}\limits_{{t^{\prime} \in \left\{ {1,2} \right\}}} \left| {v_{j} - m_{{t^{\prime}}} } \right| = t} \right\},\quad t = 1,2.$$
4. *Medoid Update*: For each cluster ${C}_{t}$ recompute the medoid as the data point that minimizes the total dissimilarity within the cluster:
  $${m}_{t}=\text{arg}\underset{{v}_{j}\in {C}_{t}}{min}\sum_{{v}_{k}\in {C}_{t}} |{v}_{j}-{v}_{k}|, t=\text{1,2}.$$
5. *Iteration*: Repeat Steps 3–4 until medoid assignments stabilize (i.e., no changes occur for three consecutive iterations).

Upon convergence, the cluster with the larger medoid ($max({m}_{1}, {m}_{2})$) is retained as the set of discriminative features, while the smaller cluster is discarded. This approach inherently adapts to the data structure: if the coefficient distribution is strongly bimodal, the clusters will separate clearly, whereas overlapping distributions result in conservative feature retention.

This integration of K-Medoids clustering with compressed sensing ensures that feature selection is both interpretable and adaptive, bridging the gap between regularization-driven sparsity and data-driven decision-making.

### Dimensionality reduction

Following the automated feature selection via K-Medoids clustering in section “Sparse coefficient selection via K-Medoids clustering”, the final step involves constructing a parsimonious classification model using only the selected biomarkers. This dimensionality reduction process addresses the inherent challenges of high-dimensional proteomics data by retaining discriminative features while eliminating noise and redundancy.

The sparse coefficient vector $\widetilde{{\varvec{\omega}}}$, derived from clustering the absolute coefficients (Section “Sparse Coefficient selection via K-Medoids clustering”), serves as the foundation for the reduced-dimensional classifier. Specifically, the decision function in section “Linear decision function” is redefined to operate exclusively on the subset of selected features:

$${f}_{\widetilde{{\varvec{\omega}}}}({{\varvec{x}}}_{i}^{(\mathcal{S})})=\sum_{k\in \mathcal{S}} {\widetilde{\omega }}_{k}{x}_{i,k},$$

where $\mathcal{S}=\left\{k|{\widetilde{\omega }}_{k}\ne 0\right\}$ denotes the indices of retained features. By discarding coefficients clustered as non-informative $({\widetilde{\omega }}_{k}=0)$, the model effectively reduces the input dimensionality from $d$ to $| \mathcal{S}|\ll d$, where $| \mathcal{S}|$ represents the number of selected biomarkers.

#### Implementation workflow

1. Feature Subset Extraction: For each sample ${{\varvec{x}}}_{i}$, only the intensities of features in $\mathcal{S}$ are retained, forming a reduced input vector ${{\varvec{x}}}_{i}^{( \mathcal{S})}\in {\mathbb{R}}^{| \mathcal{S}|}$.
2. Classifier Deployment: The linear decision boundary $\langle \widetilde{{\varvec{\omega}}},{{\varvec{x}}}^{( \mathcal{S})}\rangle =0$ partitions samples into classes using the sparse coefficient weights. New samples are classified by projecting their proteomic profiles onto the selected features and computing the decision score.

#### Key advantages of dimensionality reduction:

- Enhanced Generalization: Limiting the model to a sparse subset of features mitigates overfitting by eliminating noisy or redundant variables, which is particularly critical in $d\gg n$ settings.
- Interpretability: The reduced feature set directly corresponds to putative biomarkers, enabling biological validation and mechanistic insights.
- Computational Efficiency: Lower dimensionality accelerates prediction in clinical applications and reduces memory requirements for large-scale datasets.

This step concludes the workflow, transforming a high-dimensional proteomic dataset into a clinically actionable model that balances predictive accuracy, interpretability, and scalability. The integration of automated feature selection with dimensionality reduction ensures that the final classifier is both robust to noise and aligned with the biological parsimony expected in biomarker discovery.

## Results

### Simulation design

The synthetic proteomics data generation framework was designed to systematically evaluate feature selection robustness and classification performance under controlled conditions. To emulate functional protein modules with intrinsic biological correlations, the features were partitioned into contiguous blocks of 50 features each. Each block ${{\varvec{z}}}_{i}^{(f)}=({z}_{i,50f+1},\dots ,{z}_{i,50f+50}{)}^{\top }$ was generated from a multivariate normal distribution ${\mathcal{N}}_{50}(0,\boldsymbol{\Sigma })$, where the covariance matrix $\boldsymbol{\Sigma }$ followed an autoregressive structure of order 1. Specifically, the diagonal elements of $\boldsymbol{\Sigma }$ satisfied ${\sigma }_{ss}=1$, and the off-diagonal elements were defined as ${\sigma }_{s{s}^{\prime}}={0.8}^{\left|s-{s}^{\prime}\right|}$ for $s\ne {s}^{\prime}$. Observed features were then contaminated with technical noise to mimic mass spectrometry variability. Specifically, each feature ${x}_{i,k}$ was generated as ${x}_{i,k}={z}_{i,k}+{\epsilon }_{i,k}$, where ${\epsilon }_{i,k}\sim \mathcal{N}(0,{\sigma }_{\epsilon }^{2})$. The noise variance ${\sigma }_{\epsilon }^{2}=\frac{\text{Var}(\varvec{z})}{\text{SNR}}$ was calibrated using signal-to-noise ratios (SNR) of 100, 10, and 3, corresponding to high, medium, and low SNR regimes. The first five biomarkers ($\mathcal{K}$) were designated as ground-truth biomarkers, and each was assigned an effect size ${\beta }_{k}=1$. Finally, the binary response variable ${y}_{i}$ was generated via a logistic model: $$P({y}_{i}=1)={\left[\begin{array}{c}1+exp\left(-\sum_{k\in \mathcal{K}} {\beta }_{k}{z}_{i,k}\right)\end{array}\right]}^{-1}$$, ensuring that the simulated outcomes directly depended on the predefined discriminative features. This protocol enabled precise control over data distribution, noise levels, and biomarker effects, facilitating rigorous evaluation of method performance under diverse experimental conditions.

To rigorously ensure independence between the training and evaluation phases, a strict data partitioning strategy was implemented. The training set was generated using a fixed random seed ${S}_{\text{train}}$ correlation structure, and the set of ground-truth biomarkers. The test set, generated with a distinct random seed ${S}_{\text{test}}({S}_{\text{test}}\ne {S}_{\text{train}})$, retained identical data-generating parameters but employed fully independent noise realizations and sample draws. To prevent data leakage, the test set was strictly isolated from all stages of model training, hyperparameter tuning, and feature selection. This design, supported by seed management and separation of data generation logic, ensured experimental reproducibility and safeguarded the reliability of validation outcomes.

To rigorously assess the performance of the proposed method, four feature selection and classification approaches were implemented and compared. The proposed method, termed ST-CS, integrates 1-bit compressed sensing (section "Optimization Framework") with K-Medoids clustering (section "Sparse Coefficient Selection via K-Medoids Clustering") to automate feature selection. For benchmarking, three established methods were evaluated:

1. *Hard-Thresholded CS (HT-CS)*, which solves the same 1-bit CS optimization problem but applies a fixed posthoc threshold (0.001) to retain coefficients exceeding this value;
2. *LASSO logistic regression*, implemented via the R/glmnet package with $\lambda$ tuned through tenfold cross-validation;
3. *SPLSDA* was implemented using the R/spls package, with hyperparameters tuned via a grid search over the sparsity parameter $\eta$ (ranging from 0.1 to 0.9 in increments of 0.1) and the number of latent components $K$ (ranging from 1 to 5). The optimal combination of $\eta$ and $K$ was selected by maximizing the 5 cross-validated error classification rate on the training set.

Performance was evaluated at two complementary levels: feature selection efficacy and classification accuracy. For feature selection assessment, six metrics were computed:

1. *Sensitivity*: Proportion of true biomarkers correctly identified;
2. *Specificity*: Proportion of non-biomarkers correctly excluded;
3. *Precision*: Proportion of selected features that are true biomarkers;
4. *False Discovery Rate (FDR)*: Proportion of selected features that are false positives;
5. *F1 Score*: Harmonic mean of precision and sensitivity;
6. *Matthews Correlation Coefficient (MCC)*: Balanced measure accounting for true/false positives/negatives.

Classification performance was quantified exclusively using the Area Under the Receiver Operating Characteristic Curve (AUC) derived from the independent test set. To guarantee statistical reliability, each experimental configuration (spanning noise levels $SNR=100, 10, 3$, sample sizes $n=40-200$, and feature dimensions $d=500$) was evaluated across 1000 Monte Carlo replicates, with distinct training-test splits generated for each replicate.

### Simulation results

**Fig. 2:**
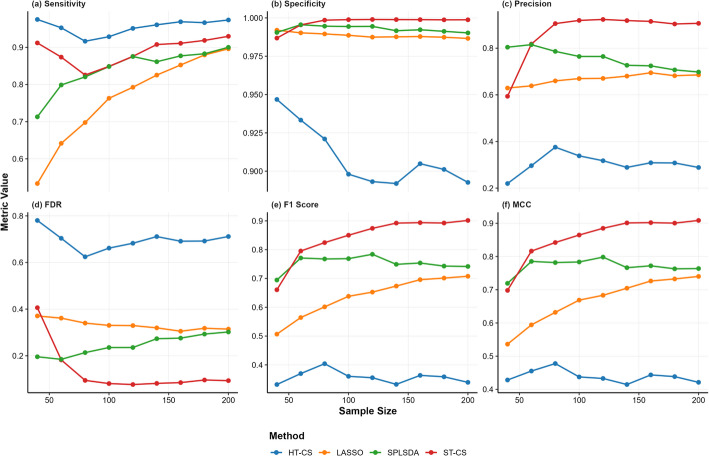
Robust Feature Selection in High SNR (SNR = 100) Simulations: Benchmarking ST-CS Against Traditional Methods Across Different Sample Sizes (n = 40–200). Line colors: HT-CS (blue), LASSO (orange), SPLSDA (green), ST-CS (red). Performance metrics (sensitivity, specificity, precision, FDR, F1 score, MCC) are plotted against sample size

**Fig. 3:**
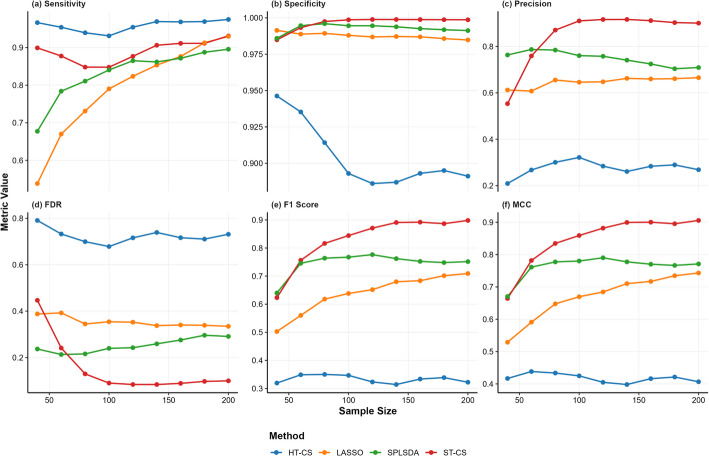
Robust Feature Selection in Medium SNR (SNR = 10) Simulations: Benchmarking ST-CS Against Traditional Methods Across Different Sample Sizes (n = 40–200). Line colors: HT-CS (blue), LASSO (orange), SPLSDA (green), ST-CS (red). Performance metrics (sensitivity, specificity, precision, FDR, F1 score, MCC) are plotted against sample size

**Fig. 4:**
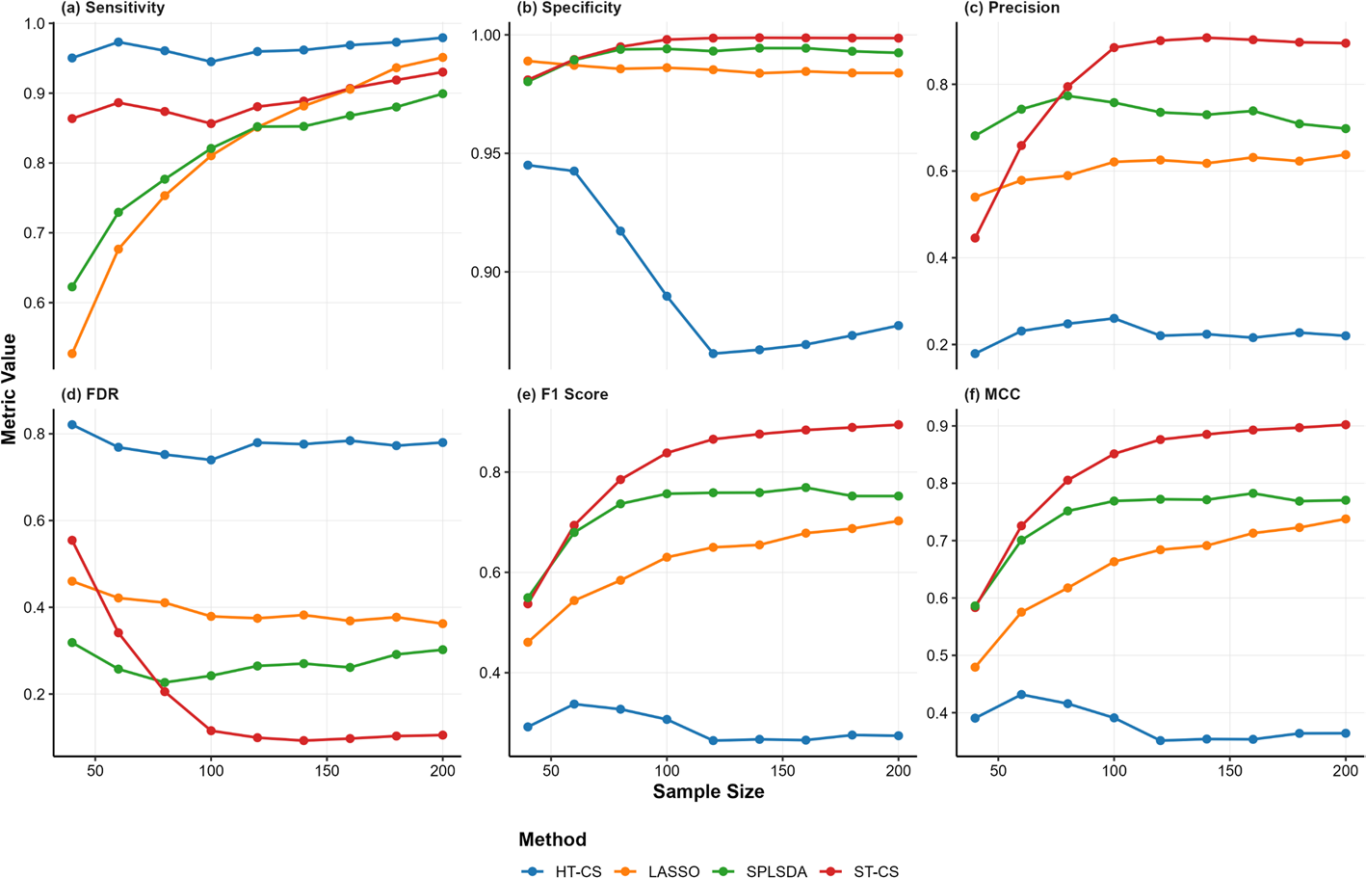
Robust Feature Selection in Low SNR (SNR = 3) Simulations: Benchmarking ST-CS Against Traditional Methods Across Different Sample Sizes (n = 40–200). Line colors: HT-CS (blue), LASSO (orange), SPLSDA (green), ST-CS (red). Performance metrics (sensitivity, specificity, precision, FDR, F1 score, MCC) are plotted against sample size

Figures 2, 3, 4 and Table 1 show the simulation results across high, medium, and low SNR conditions demonstrate distinct performance patterns among the evaluated methods (ST-CS, HT-CS, LASSO, and SPLSDA). Below is a structured synthesis of the key findings:

1. *Sensitivity*: HT-CS consistently achieved the highest sensitivity across all $n$ and SNR levels, attributed to its fixed thresholding strategy that retains weak discriminative features. However, this came at the cost of inflated false positives. ST-CS, while slightly less sensitive than HT-CS, maintained competitive sensitivity ($>80\%$) by adaptively separating true biomarkers from noise via K-Medoids clustering. LASSO exhibited the lowest sensitivity, especially at small n ($<60\%$ for $n=40$), due to excessive sparsity from ${{\ell}}_{1}$ regularization.
2. *Specificity*: ST-CS achieved near-perfect specificity ($>99.8\%$ at larger n), significantly outperforming HT-CS, which struggled with false positives (specificity $<95\%$). LASSO and SPLSDA showed moderate specificity but failed to match ST-CS’s precision in noise exclusion.
3. *Precision and FDR*: ST-CS achieved the highest precision ($>90\%$ at $n>100$), driven by its ability to minimize false positives. HT-CS had the lowest precision due to its fixed threshold, while LASSO and SPLSDA showed intermediate precision but lagged behind ST-CS. ST-CS reduced FDR by $20-50\%$ compared to HT-CS, particularly at large $n$, as clustering refined feature selection.
4. *Balanced Metrics (F1 Score and MCC)*: ST-CS outperformed all methods in F1 score and MCC, demonstrating its ability to harmonize sensitivity and precision. HT-CS’s high sensitivity but extremely low precision resulted in the worst F1 score ($<40\%$) and MCC ($<50\%$), while LASSO and SPLSDA showed moderate values.
5. *Classification performance (AUC)*: ST-CS demonstrated robust classification performance across varying SNR and $n$, with distinct comparative advantages over HT-CS, LASSO, and SPLSDA. Under high and medium SNR conditions, ST-CS achieved marginally higher AUC values than HT-CS. This is because HT-CS’s reliance on fixed thresholds introduced minor overfitting, slightly reducing its generalizability despite comparable sensitivity. At low SNR, ST-CS and HT-CS exhibited similar AUC performance, as both methods faced challenges in distinguishing weak signals from noise. LASSO and SPLSDA consistently underperformed across all SNR levels: LASSO’s excessive sparsity discarded critical biomarkers, while SPLSDA’s retention of redundant features introduced noise into classification models.

**Table 1:** Comparing AUC (%) of ST-CS and traditional methods across different sample sizes in varied SNR simulations

SNR	Sample size	ST-CS	HT-CS	LASSO	SPLSDA
High	40	**82.73**	81.79	78.92	82.24
	60	**85.53**	85.07	82.03	84.11
	80	**85.76**	85.54	83.47	84.35
	100	**85.79**	85.77	83.94	84.31
	120	**86.34**	86.24	84.60	84.80
	140	**86.60**	86.37	85.10	84.55
	160	**86.51**	86.30	85.28	84.79
	180	**86.60**	86.38	85.60	84.78
	200	**86.88**	86.58	85.91	85.12
Medium	40	**81.42**	80.66	77.23	80.28
	60	**84.46**	83.93	80.89	82.76
	80	**84.74**	84.59	82.37	83.42
	100	**84.80**	84.71	82.74	83.24
	120	**85.35**	85.31	83.51	83.83
	140	**85.59**	85.51	84.07	83.77
	160	**85.57**	85.42	84.19	83.69
	180	**85.62**	85.53	84.52	83.88
	200	**85.81**	85.61	84.90	84.13
Low	40	**78.25**	77.70	72.97	76.09
	60	**81.57**	81.41	77.44	79.79
	80	**82.85**	82.61	79.46	80.93
	100	82.61	**82.66**	80.09	80.94
	120	83.25	**83.32**	80.95	81.63
	140	83.40	**83.52**	81.60	81.65
	160	83.32	**83.39**	81.80	81.64
	180	83.53	**83.57**	82.26	81.85
	200	83.77	**83.80**	82.71	82.11

Bold values indicate the highest AUC among all methods for a given SNR and sample size

In summary, ST-CS outperformed existing methods in specificity, precision, F1 score, MCC, and AUC, particularly at larger sample sizes and higher SNR. HT-CS prioritized sensitivity at the cost of specificity, while LASSO and SPLSDA offered intermediate performance but lacked the balanced efficiency of ST-CS. These results highlight ST-CS’s robustness in automating feature selection while maintaining classification accuracy across diverse SNR conditions.

### Application to real data

To validate the practical utility of the proposed method, we evaluated its performance on three independent proteomic datasets from the Clinical Proteomic Tumor Analysis Consortium (CPTAC): PDC000356 (intrahepatic cholangiocarcinoma) [11], PDC000446 (glioblastoma) [12], and PDC000362 (ovarian serous cystadenocarcinoma) [13]. All datasets were preprocessed using variance filtering (retaining features with variance > 0.8 times the maximum) and standardized. For rigorous evaluation, a fivefold cross-validation framework was implemented, where each dataset was randomly partitioned into training and testing subsets. Model performance was quantified by the average AUC, while feature sparsity was assessed by the mean number of selected biomarkers.

*PDC000356 Dataset:* This dataset comprised 205 samples (101 tumors vs. 104 normal tissues) with 925 protein features retained after preprocessing. As shown in Table 2, ST-CS achieved the highest average AUC of 97.47%, matching HT-CS (97.47%) but with 57% fewer selected features (37 ± 12 vs. 86 ± 19). LASSO yielded suboptimal performance (AUC = 95.71%) due to excessive sparsity (12 ± 9 features), while SPLSDA retained a larger number of features (412 ± 191) despite comparable AUC (97.02%). These results highlight ST-CS’s unique ability to balance predictive accuracy with feature parsimony.

**Table 2:** Comparative performance of feature selection methods on CPTAC proteomic datasets: AUC and feature sparsity

Model	PDC000356		PDC000446		PDC000362	
	AUC (%)	N	AUC (%)	N	AUC (%)	N
ST-CS	97.47	37 ± 12	72.71	30 ± 11	75.86	24 ± 5
HT-CS	97.47	86 ± 19	72.15	58 ± 6	75.61	59 ± 9
LASSO	95.71	12 ± 9	67.80	22 ± 16	61.00	18 ± 9
SPLSDA	97.02	412 ± 191	71.38	505 ± 408	70.75	591 ± 251

N is the number of features selected (mean ± standard deviation)

*PDC000446 Dataset*: For glioblastoma proteomics (25 recurrent vs. 102 primary tumors, 1,753 features retained), ST-CS demonstrated superior performance. It achieved an AUC of 72.71%, outperforming HT-CS (72.15%), LASSO (67.80%), and SPLSDA (71.38%). Notably, ST-CS selected only 30 ± 11 features, nearly half the number required by HT-CS (58 ± 6) and far fewer than SPLSDA (505 ± 408).

*PDC000362 Dataset*: The ovarian serous cystadenocarcinoma dataset included 64 samples (45 primary tumors vs. 19 metastatic tumors) with 1,375 protein features after preprocessing. ST-CS achieved an AUC of 75.86%, surpassing HT-CS (75.61%), LASSO (61.00%), and SPLSDA (70.75%) and retaining only 24 ± 5 features. This performance further validates ST-CS’s adaptability to diverse cancer types and its ability to eliminate redundancy without sacrificing classification accuracy.

*Biological Validation*: To assess the biological significance of features selected by ST-CS, we conducted literature validation and pathway enrichment analysis on the CPTAC datasets. For intrahepatic cholangiocarcinoma (PDC000356), key proteins such as SULT1A1 were identified as tumor suppressors linked to chemoresistance and sulfonation-driven metabolic regulation [14] (Supplementary Table S1). In glioblastoma (PDC000446), BIN1 emerged as a critical tumor suppressor epigenetically silenced in gliomas [15] (Supplementary Table S2). For ovarian serous cystadenocarcinoma (PDC000362), LRRC15 was validated as a metastasis-associated protein with significant overexpression in intestinal metastases [16] (Supplementary Table S3). Gene Ontology (GO) and Kyoto Encyclopedia of Genes and Genomes (KEGG) pathway analyses revealed that ST-CS-selected features were enriched in disease-relevant processes. For example, intrahepatic cholangiocarcinoma biomarkers were significantly associated with peroxisome metabolism (KEGG: hsa04146, FDR = 1.37e−05), a pathway implicated in oxidative stress and lipid dysregulation (Supplementary Figure S1). Glioblastoma features highlighted purine ribonucleotide metabolism (GO:0009150, FDR = 0.0021), aligning with known metabolic adaptations in aggressive tumors. These findings underscore the functional relevance of ST-CS-selected biomarkers to disease mechanisms.

## Discussion

This study introduces a novel hybrid framework for automated sparse feature selection in high-dimensional proteomics data, integrating 1-bit compressed sensing with K-Medoids clustering to address critical limitations of existing methodologies. The proposed approach, termed ST-CS, operates through three core components: (1) a dual-regularized optimization framework enforcing simultaneous sparsity and stability via explicit ${\ell}_{1}$- and ${\ell}_{2}$-norm constraints, (2) data-driven feature selection via K-Medoids clustering on coefficient magnitudes to automate threshold determination, and (3) construction of a parsimonious classifier using only the retained biomarkers.

The key theoretical innovation lies in replacing manual thresholding in conventional 1-bit CS with K-Medoids clustering, which introduces a data-adaptive mechanism for distinguishing true biomarkers from noise. The algorithm objectively partitions features without subjective cutoff tuning by leveraging the inherent bimodal distribution of optimized coefficients—where discriminative features exhibit magnitudes significantly deviating from near-zero noise. This is mathematically grounded in minimizing intra-cluster Manhattan distances, ensuring robustness to outliers and adaptability to varying signal-to-noise regimes.

In our simulation framework, several parameters were meticulously designed to replicate the complexity of real-world proteomics data while retaining controlled experimental conditions. First, to emulate the modular architecture of biological systems, features were partitioned into contiguous blocks of 50 variables, mirroring co-regulated protein clusters commonly observed in functional pathways or complexes [17]. Within each block, an autoregressive correlation structure was implemented, where the covariance between features decayed exponentially with distance. This design mimics the weakening interaction strength observed in biological networks as molecular or functional proximity decreases [18]. To simulate the sparse nature of biomarker discovery, only five causal features—strategically assigned to the first five positions within the initial block—were directly linked to the binary outcome [19]. These biomarkers retained the same decaying correlation pattern as their block, reflecting real-world scenarios where disease-associated proteins often reside within tightly coordinated functional modules [20]. Technical variability, a hallmark of mass spectrometry data, was introduced by contaminating observed features with additive Gaussian noise, calibrated to predefined signal-to-noise ratios (SNR = 100, 10, 3) [21]. Crucially, outcome variables were generated using the noise-free protein intensities rather than the noise-contaminated measurements, ensuring that the simulated disease labels exclusively reflected true biological signals rather than technical artifacts. This deliberate separation of signal and noise preserved the causal integrity of biomarker-outcome associations, a cornerstone for validating feature selection accuracy. Collectively, these settings—modular block architecture, biologically plausible correlation decay, sparse causal features, and SNR-calibrated technical noise—establish a synthetic yet ecologically valid framework that captures the high dimensionality, sparsity, and technical variability inherent to proteomic studies, thereby rigorously benchmarking method performance under realistic conditions.

The comparative analysis of simulation outcomes elucidates fundamental distinctions in the operational mechanisms and inherent limitations of the four evaluated methods. HT-CS, relying on fixed posthoc thresholding, prioritizes sensitivity by retaining weakly discriminative features, yet its rigidity introduces substantial false positives, particularly in high-dimensional settings where noise dominates. While this approach ensures broad biomarker capture, its inability to adaptively suppress noise undermines specificity and precision. LASSO, governed by aggressive ${\ell}_{1}$ regularization, enforces excessive sparsity, discarding critical biomarkers—a limitation magnified in small-sample proteomics studies. Its reliance on cross-validated $\lambda$ tuning further amplifies instability under low SNR regimes [22]. SPLSDA, though effective in handling multicollinearity, retains redundant features due to its latent variable construction, thereby diluting biomarker specificity and inflating model complexity [23]. In contrast, ST-CS uniquely integrates 1-bit compressed sensing with K-Medoids clustering, replacing subjective thresholds with data-driven separation of signal and noise. By exploiting the bimodal distribution of coefficient magnitudes, ST-CS dynamically partitions features into high-confidence biomarkers and irrelevant variables, achieving superior specificity (> 99.8%) without sacrificing sensitivity (> 80%). This dual regularization—${\ell}_{1}$ for sparsity and ${\ell}_{2}$ for stability—coupled with automated clustering, ensures robust performance across SNR levels (AUC: 78.25–86.88% vs. HT-CS: 77.70–86.58%) [9].

The validation on real-world proteomic datasets further substantiates the practical superiority of ST-CS in biomarker discovery. For the PDC000356 dataset (intrahepatic cholangiocarcinoma), ST-CS achieved equivalent AUC to HT-CS (97.47%) but with 57% fewer selected features (37 vs. 86), demonstrating its unique ability to eliminate redundant variables while preserving predictive power. This aligns with the simulation findings where HT-CS’s reliance on fixed thresholds retained noise-driven coefficients, inflating false positives. In contrast, ST-CS’s data-driven clustering adaptively discarded non-discriminative features, thereby enhancing specificity without compromising sensitivity. Similarly, for the glioblastoma dataset (PDC000446), ST-CS outperformed HT-CS in AUC (72.71% vs. 72.15%) while selecting nearly half the number of biomarkers (30 vs. 58), underscoring its robustness in low-sample-size scenarios. The stark contrast with LASSO—which exhibited suboptimal AUC (67.80%) due to excessive sparsity—highlights ST-CS’s balanced regularization strategy. LASSO discarded critical biomarkers through aggressive ${\ell}_{1}$ shrinkage, and ST-CS retained biologically meaningful features via dual ${\ell}_{1}$ and ${\ell}_{2}$ constraints and automated clustering. SPLSDA, though competitive in AUC (71.38%), retained a larger number of features (505 ± 408) with higher variability in feature selection (standard deviation = 408), reflecting its inherent limitation in handling multicollinearity through latent variables [23]. In contrast, ST-CS’s stability in feature selection, characterized by low variability (standard deviation = 11), combined with its parsimonious selection of biomarkers (30 features), ensures both reproducibility and clinical feasibility. Collectively, these results validate that ST-CS successfully bridges the gap between sensitivity and specificity: it leverages compressed sensing for sparse signal recovery while dynamically calibrating feature retention through clustering, yielding parsimonious yet accurate models. This dual advantage—superior generalizability and biological interpretability—positions ST-CS as a robust tool for translational proteomics, where minimizing false discoveries and maximizing clinical actionability are paramount.

The ST-CS framework, while primarily validated in proteomics, demonstrates inherent adaptability to other high-dimensional omics data characterized by sparsity and technical noise. For instance, transcriptomic datasets often exhibit challenges such as feature redundancy and weak signal-to-noise ratios in large-scale gene expression studies. ST-CS’s dual regularization (${\ell}_{1}$ for sparsity and ${\ell}_{2}$ for stability) could dynamically prioritize critical transcripts linked to disease phenotypes while suppressing non-informative noise, mirroring its success in proteomic biomarker discovery. Similarly, metabolomic studies face persistent issues with spectral noise in untargeted profiling. ST-CS’s clustering-driven thresholding mechanism may enhance specificity by prioritizing metabolic features with higher discriminative power, even in noisy environments. The framework’s adaptability allows potential extension to multi-omics integration, where joint modeling of proteomic, transcriptomic, and metabolomic features could uncover cross-modal regulatory networks. The theoretical foundation of compressed sensing supports its potential applicability across diverse omics domains, including transcriptomics and metabolomics, where high-dimensional data and noise are prevalent.

### Limitations and future directions

Despite its promising performance, this study has several limitations that warrant consideration. First, the simulation framework assumes predefined block-wise correlations and autoregressive covariance structures, which may oversimplify the intricate topological organization of real biological networks. Future work could incorporate more heterogeneous correlation patterns, such as scale-free or small-world networks, to better emulate complex protein interactions. Second, the current implementation relies on sequential quadratic programming via Rdonlp2, which exhibits nonlinear runtime scaling for ultra-high-dimensional datasets (e.g., > 10,000 features), as demonstrated by a tenfold runtime increase from $d={8,000}$ (618 s) to $d={12,000}$ (6,423 s) in Supplementary Table S4. While memory usage grows linearly with $d$ (335.8 MB at $d={12,000}$), the iterative optimization framework of Rdonlp2 becomes computationally prohibitive for $d>{10,000}$. Future work will prioritize replacing Rdonlp2 with first-order methods (e.g., proximal gradient descent) to reduce time complexity and integrating GPU-accelerated sparse matrix operations to enhance scalability. Additionally, ST-CS is currently tailored for binary classification; extending it to multiclass settings would broaden its applicability in multi-disease diagnostics. Addressing these limitations will refine the method’s robustness, scalability, and versatility, positioning it as a cornerstone for precision biomarker discovery.

## Electronic supplementary material

Below is the link to the electronic supplementary material.

Supplementary Material 1

## Data Availability

This study used both simulated produced data and real-world data. To ensure the reproducibility of the simulated data and the accuracy of real-world data analysis, all original codes are available at [https://github.com/wenfudong/ST-CS.git]. This paper analyzes existing real-world data, publicly available at the Clinical Proteomic Tumor Analysis Consortium (CPTAC) via identifiers PDC000356, PDC000446, and PDC000362 [https://proteomics.cancer.gov/programs/cptac].

## Abbreviations

LASSO: Least absolute shrinkage and selection operator
SPLSDA: Sparse partial least squares discriminant analysis
CS: Compressed sensing
ST-CS: Soft-thresholded compressed sensing
HT-CS: Hard-thresholded compressed sensing
SNR: Signal-to-noise ratio
FDR: False discovery rate
MCC: Matthews Correlation Coefficient
AUC: Area under the receiver operating characteristic curve
CPTAC: Clinical proteomic tumor analysis consortium
GO: Gene ontology
*KEGG*: Kyoto encyclopedia of genes and genomes
