# Supplementary material for: Automated sparse feature selection in high-dimensional proteomics data via 1-bit compressed sensing and K-Medoids clustering
Source: BMC Bioinformatics. 2025 Jul 1;26:165. doi: 10.1186/s12859-025-06193-2 (PMC12220089; doi:10.1186/s12859-025-06193-2)
Supplement: Supplementary file 1 — Supplementary Material 1 [file 12859_2025_6193_MOESM1_ESM.pdf]

Supplementary Table S1. ST-CS Selected Proteins in Intrahepatic Cholangiocarcinoma (CPTAC PDC000356): Cross-Validation Frequency (N)

Protein	N	Protein	N	Protein	N	Protein	N	Protein	N
AADAT	5	ACSM3	4	ACMSD	3	ACAA1	2	ACSL1	1
ACSM2A	5	ANGPTL3	4	ACSM1	3	ACBD4	2	ALDH7A1	1
ACSM2B	5	CLEC4G	4	EXOC3L4	3	AK4	2	AS3MT	1
ECI2	5	CYB5A	4	S100A11	3	ALDH4A1	2	ASGR1	1
GAS2	5	DDT	4	SELENBP1	3	CES1	2	ASGR2	1
GLS2	5	FABP1	4	SULT1A3	3	ETFRF1	2	BDH1	1
MMUT	5	LDHD	4			FXYD1	2	CAPG	1
NDRG2	5	PALMD	4			GLYCTK	2	CLEC4M	1
RMDN2	5	STBD1	4			MMAB	2	CYP2C9	1
SCP2	5					PCK2	2	CYP2E1	1
SULT1A1	5					PIPOX	2	DHRS11	1
SULT1A2	5					REEP6	2	ESPN	1
SULT2A1	5					RIDA	2	HAO1	1
TKFC	5					S100A6	2	LAMB1	1
TTC36	5					TFR2	2	LYVE1	1
								OIT3	1
								PAH	1
								PGRMC1	1
								PHYH	1
								PLIN5	1
								PPP1R1A	1
								PVALB	1
								SPINT1	1
								VSNL1	1

N is the number of times the protein is selected in the five-fold cross-validation.

Supplementary Table S2. ST-CS Selected Proteins in Glioblastoma (CPTAC PDC000446): Cross-Validation Frequency (N)

Protein	N	Protein	N	Protein	N	Protein	N	Protein	N
ADAP1	5	ATP5IF1	4	ARRB1	3	ADCY5	2	ACO2	1
ATP5F1E	5	DLG2	4	AUH	3	AK5	2	ALDH2	1
BIN1	5	FTL	4	EFHD1	3	ASAH1	2	ATP1B1	1
GOT2	5	NDUFA2	4	MAP6D1	3	ATP6V1G2	2	ATP6V1D	1
TINAGL1	5			NAPEPLD	3	CPNE6	2	ATP6V1E1	1
				PDE1A	3	FBXO2	2	ATP8A1	1
						HDAC11	2	CAMKK1	1
						IQSEC2	2	CARNS1	1
						MAD2L1	2	CD55	1
						MBLAC2	2	CD93	1
						MYO1D	2	CEP55	1
						PCSK2	2	ENPP2	1
						PLCL1	2	FAAH	1
						PLEKHA1	2	FTH1	1
						PPM1H	2	GJA1	1
						RBP7	2	GLS	1
						SEPT4	2	HK1	1
						SIGLEC1	2	HSPA12A	1
						STXBP6	2	IDH3A	1
						SUCLA2	2	IGSF8	1
						TPRG1L	2	IQSEC1	1
								JAM3	1
								KCNAB2	1
								LYNX1	1
								LYRM1	1
								LYRM9	1
								ME3	1
								MKI67	1
								NCAPD2	1
								NECAB1	1
								NQO1	1
								NSF	1
								OGDHL	1
								PCNA	1
								PCSK1N	1
								PEX5L	1
								PIP4K2A	1
								PODXL	1
								PRKAR2B	1
								PRODH	1
								RASGRF2	1
								RGS14	1
								SH3GLB2	1
								SMC2	1
								TGIF1	1
								TMEM167A	1
								TPPP	1

N is the number of times the protein is selected in the five-fold cross-validation.

Supplementary Table S3. ST-CS Selected Proteins in Ovarian Serous Cystadenocarcinoma (CPTAC PDC000362): Cross-Validation Frequency (N)

Protein	N	Protein	N	Protein	N	Protein	N	Protein	N
GPD1	5	ACSL1	4	COPZ2	3	APBB1IP	2	ARHGAP30	1
LIMD2	5	CD36	4	CORO1A	3	CILP	2	ARHGDIB	1
LRRC15	5	FABP4	4	HCLS1	3	COL11A1	2	CAPG	1
NNMT	5	SVEP1	4	IL16	3	CTSH	2	CNN2	1
PLIN1	5			RGCC	3	EVL	2	COL5A2	1
TMEM119	5			RTN1	3	FBLN2	2	COLEC11	1
						FKBP11	2	COMP	1
						FN1	2	CPA3	1
						LCP1	2	CTHRC1	1
						LPXN	2	FAP	1
						MGST1	2	FHL3	1
						MXRA5	2	FKBP14	1
						POSTN	2	GIMAP2	1
						PYCARD	2	ICAM1	1
						RBP1	2	ITGBL1	1
						S100A11	2	LCP2	1
								LSP1	1
								MMP11	1
								MT1H	1
								PPL	1
								RAC2	1
								SASH3	1
								TCEAL4	1
								TPSAB1	1
								TSPAN8	1
								VCAN	1

N is the number of times the protein is selected in the five-fold cross-validation.

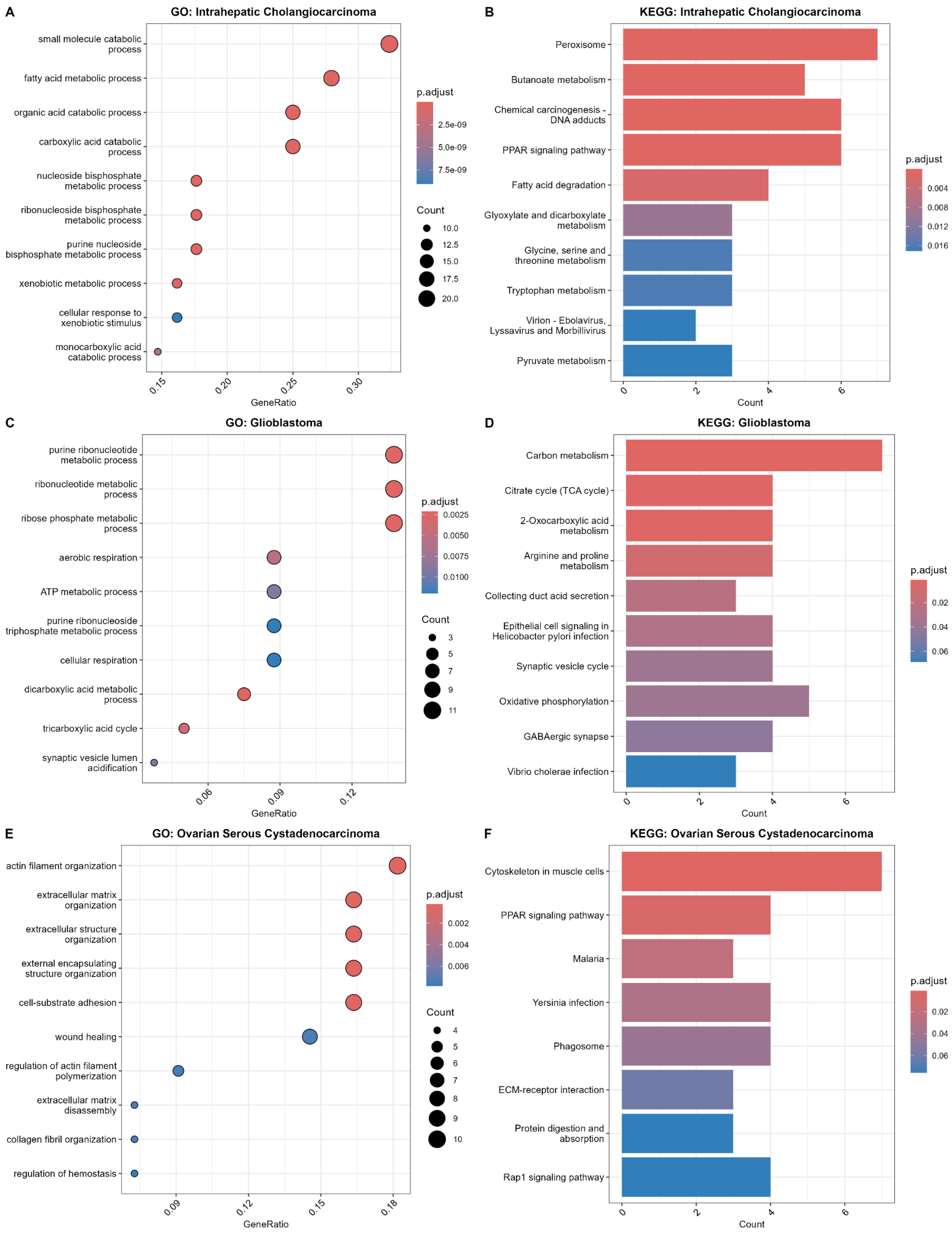

Supplementary Figure S1. Integrated Functional Enrichment Analysis of ST-CS-Selected Proteins in Intrahepatic Cholangiocarcinoma, Glioblastoma, and Ovarian Serous Cystadenocarcinoma

(A) GO biological process enrichment for intrahepatic cholangiocarcinoma (CPTAC PDC000356). (B) KEGG pathway enrichment for intrahepatic cholangiocarcinoma. (C) GO biological process enrichment for glioblastoma (CPTAC PDC000446). (D) KEGG pathway enrichment for glioblastoma. (E) GO biological process enrichment for ovarian serous cystadenocarcinoma (CPTAC PDC000362). (F) KEGG pathway enrichment for ovarian serous cystadenocarcinoma. Proteins with cross-validation frequency N⩾1 were analyzed. Dot plots (GO) show the top 10 enriched terms sorted by adjusted p-value; bar plots (KEGG) display pathways ranked by enrichment factor.

Supplementary Table S4. Runtime and Memory Usage of Sequential Quadratic Programming via Rdonlp2 Across Increasing Feature Dimensions

Number of features	Run time (seconds)	Memory usage (MB)
2000	417	30.64
4000	465	91.66
6000	300	152.69
8000	618	213.73
10000	3996	274.77
12000	6423	335.80

Data Description: Evaluated feature dimensions ranging from 2,000 to 12,000 under a fixed sample size n=200. Data generation followed the protocol in Section 3.1 (autoregressive block-wise correlations, SNR = 100).

Hardware: Benchmarks were conducted on a desktop with 16 GB RAM (Intel i7-11700K CPU, NVIDIA RTX 3060 Ti GPU).

Software: Implemented in R 4.3.1 using the Rdonlp2 solver.
